# The Overlapping and Distinct Roles of HAM Family Genes in *Arabidopsis* Shoot Meristems

**DOI:** 10.3389/fpls.2020.541968

**Published:** 2020-09-04

**Authors:** Han Han, Yuan Geng, Lei Guo, An Yan, Elliot M. Meyerowitz, Xing Liu, Yun Zhou

**Affiliations:** ^1^Department of Botany and Plant Pathology, Purdue University, West Lafayette, IN, United States; ^2^Purdue Center for Plant Biology, Purdue University, West Lafayette, IN, United States; ^3^Division of Biology and Biological Engineering, California Institute of Technology, Pasadena, CA, United States; ^4^Howard Hughes Medical Institute, California Institute of Technology, Pasadena, CA, United States; ^5^Department of Biochemistry, Purdue University, West Lafayette, IN, United States

**Keywords:** shoot development, *Arabidopsis*, HAIRY MERISTEM, stem cells, confocal imaging, shoot apical meristems

## Abstract

In *Arabidopsis* shoot apical meristems (SAMs), a well-characterized regulatory loop between WUSCHEL (WUS) and CLAVATA3 (CLV3) maintains stem cell homeostasis by regulating the balance between cell proliferation and cell differentiation. WUS proteins, translated in deep cell layers, move into the overlaying stem cells to activate *CLV3*. The secreted peptide CLV3 then regulates *WUS* levels through a ligand-receptor mediated signaling cascade. *CLV3* is specifically expressed in the stem cells and repressed in the deep cell layers despite presence of the WUS activator, forming an apical-basal polarity along the axis of the SAM. Previously, we proposed and validated a hypothesis that the HAIRY MERISTEM (HAM) family genes regulate this polarity, keeping the expression of *CLV3* off in interior cells of the SAM. However, the specific role of each individual member of the HAM family in this process remains to be elucidated. Combining live imaging and molecular genetics, we have dissected the conserved and distinct functions of different HAM family members in control of *CLV3* patterning in the SAMs and in the *de novo* shoot stem cell niches as well.

## Introduction

Pluripotent stem cells in plant shoot apical meristems (SAMs) can continuously divide and initiate new leaves and flowers. In the model plant *Arabidopsis*, the stem cells are located at the apical tip of the SAM, while cells that help to specify the stem cells are located more basally (Meyerowitz, 1997). Along the axis of the SAM, a regulatory feedback loop involving CLAVATA3 (CLV3) and WUSCHEL (WUS) controls stem cell homeostasis through cell-cell communication between these two cell types (Laux et al., 1996; Mayer et al., 1998; Fletcher et al., 1999; Brand et al., 2000; Schoof et al., 2000). *CLV3* mRNAs are specifically expressed in the stem cells at the central zone but not expressed in the rib meristem cells that are located beneath the stem cells (Fletcher et al., 1999; Brand et al., 2000). In contrast, *WUS* transcripts are confined to a small group of cells in the rib meristem that has been defined as the organizing center (OC) (Mayer et al., 1998; Schoof et al., 2000). Through plasmodesmata, WUS protein, a homeodomain transcription factor, can move from the cells at the OC into the stem cells in the central zone (Yadav et al., 2011; Daum et al., 2014), where it can directly activate *CLV3* expression (Yadav et al., 2011). *CLV3* encodes a secreted peptide that activates a ligand-receptor mediated signaling pathway (Clark et al., 1997; Fletcher et al., 1999; Kinoshita et al., 2010; Nimchuk et al., 2011; Nimchuk et al., 2015) to negatively regulate *WUS* levels and to limit stem cell proliferation. Thus, *WUS* and *CLV3* form a negative feedback loop to maintain stem cell homeostasis (Schoof et al., 2000).

In previous work (Zhou et al., 2015; Zhou et al., 2018), we proposed that HAIRY MERISTEM (HAM, also known as LOST MERISTEMS – LOM, Schulze et al., 2010) family transcription factors regulate the *CLV3*-*WUS* feedback loop. HAM proteins together with WUS determine the apical-basal polarity of *CLV3* expression in *Arabidopsis* SAMs and confine the *CLV3* domain to the stem cells (Han et al., 2020a). Specifically, WUS protein moves upward and activates *CLV3* in the central zone (Yadav et al., 2011; Daum et al., 2014), while HAM family members keep *CLV3* off in the rib meristem by preventing WUS-dependent activation of *CLV3* and/or repressing *CLV3* transcription (Zhou et al., 2018). This hypothesis is supported by experimental results and shown plausible by a computational model (Zhou et al., 2018). The hypothesis also aligns with a number of results from earlier, independent studies (Brand et al., 2000; Schoof et al., 2000; Brand et al., 2002; Graf et al., 2010; Schulze et al., 2010; Biedermann and Laux, 2018; Zhou et al., 2018), including a computational model that features an essential role for HAM in control of *CLV3* patterns (Gruel et al., 2018). Additionally, the concentration gradient of HAM has been shown to be essential in determining the *CLV3* domain in both well-established SAMs and in initiating axillary stem cell niches (Biedermann and Laux, 2018; Zhou et al., 2018), suggesting the important roles of HAM family genes controlling both initiation and maintenance of patterns of gene expression in plant stem cell niches.

To date, the potentially overlapping and distinct roles of HAM family members in control of *CLV3* patterning and meristem development remain unexplored. There are four HAM genes in *Arabidopsis*, which fall into two distinct subgroups—Type I and Type II (Engstrom et al., 2011). HAM1, HAM2, and HAM3 belong to the Type II clade, whereas HAM4 belongs to the Type I clade (Engstrom et al., 2011). Among them, HAM1 and HAM2 are the most closely related homologs (Schulze et al., 2010; Engstrom et al., 2011), and both HAM1 and HAM2 proteins physically interact with WUS as interacting cofactors (Zhou et al., 2015). Differently from HAM1-3 in the Type II clade that share similar N-terminal regions, the N terminus of HAM4 in the Type I clade is less similar to that in HAM1-3 (Engstrom et al., 2011). The transcripts of the Type II genes (*HAM1-3*) are targeted by the microRNA171, while the transcript of the Type I gene *HAM4* lacks the microRNA171 target site (Engstrom et al., 2011). In addition, *CLV3* is ectopically expressed in the rib meristem when all of the three Type II clade genes are nonfunctional in *Arabidopsis*, i.e. in the *ham123* triple loss of function mutant (Schulze et al., 2010; Zhou et al., 2018). In contrast, *HAM4* is specifically expressed in the provascular/vascular tissues (Zhou et al., 2015), and thus it is unlikely to be directly involved in *CLV3* patterning at the center of the SAM. These findings suggest that *HAM1*, *HAM2* and *HAM3* in the Type II clade are potentially involved in *CLV3* regulation. In this study, we aimed to define which member(s) of this clade is required and/or sufficient to control the *CLV3* patterning and therefore meristem development. We performed confocal microscope live imaging and molecular genetic analyses. Our results demonstrate that HAM1 and HAM2, both expressed in the rib meristem, are necessary and sufficient to shape the *CLV3*-expression domain in established SAMs and in *de novo*-initiated axillary meristems. In contrast, HAM3 protein, naturally expressed only in the boundary between the meristem and primordia and a few cells of the peripheral zone of the SAM, does not contribute to *CLV3* patterning. When the HAM3 protein is expressed in the HAM2 domain, it is able to shape the *CLV3* expression pattern. These results uncover the different patterns and conserved functions of the Type II HAM proteins in *Arabidopsis*, and they suggest that HAM regulates *CLV3* patterns cell-autonomously.

## Materials and Methods

### Plant Materials and Growth Conditions

The *ham123* triple mutant (Zhou et al., 2018) and *ham12* double mutant (Engstrom et al., 2011) were previously described. The *MIR171* overexpression lines were described previously (Zhou et al., 2018).

For **Figures 4A–C**, SAMs from L*er*, *ham123*, and the *pHAM1::YPET-HAM1* in *ham123* transgenic line at 27 days after germination (DAG) were sampled and analyzed at the same time using an identical procedure. For **Figures S1A, B** **Figure 4D**, SAMs from L*er*, *ham123*, and the *pHAM2::YPET-HAM2* in *ham123* transgenic line at 27 DAG were sampled and analyzed at the same time using an identical procedure. For **Figures S2A, B** and **Figure 4E**, SAMs from L*er*, *ham123*, and the *pHAM3::YPET-HAM3* in *ham123* transgenic line at 27 DAG were sampled and analyzed at the same time using an identical procedure. For **Figure 4F** and **Figures S3A–E**, SAMs from *ham123* and *ham12* at 27 DAG were sampled and analyzed at the same time using an identical procedure. For **Figures 5G–I**, SAMs from L*er*, *ham123*, and the *pHAM2::YPET-HAM3* in *ham123* transgenic line at 27 DAG were sampled and analyzed at the same time using an identical procedure. For **Figure S4**, the vegetative shoot apices including developing AMs from L*er* and the MIR171 OE transgenic line at 27 DAG were sampled and analyzed at the same time using an identical procedure. For **Figure 7**, shoot apices including developing AMs from L*er*, *ham123*, the *pHAM1::YPET-HAM1* in *ham123*, the *pHAM2::YPET-HAM2* in *ham123*, and the *pHAM3::YPET-HAM3* in *ham123* plants were grown in short days and analyzed using an identical procedure.

### Constructs and Transgenic Plants

To generate the *pHAM1::YPET-HAM1* in *ham123*, a YPET-HAM1 fusion was generated using overlapping PCR with the primers 5’- TACCGAGGGTATGAATGAATTGTACAAAAAATCTAGAATGCCCTTATCCTTTGAAAGGTTTCAAG - 3’, 5’- CTTGAAACCTTTCAAAGGATAAGGGCATTCTAGATTTTTTGTACAATTCATTCATACCCTCGGTA - 3’, 5’- CACCATGGCTGCAGCCAAGGGCGAGG - 3’, and 5’- CTAACATTTCCAAGCAGAGACAGTAACAAGT - 3’ following the published procedure (Heckman and Pease, 2007). A 3076 bp HAM1 promoter was PCR amplified using the primers 5’- ACAAgcggccgcGTTTTATATTTCAACTATCCCTAGATTTTAGC - 3’ and 5’- ACAAgcggccgcCGCCTCCTCAACAACACAGAGTAACTGTAAAAACA - 3’ (restriction enzyme sites are in lower case), and cloned to the 5’ of the YPET-HAM1 DNA fragment. A 1622 bp HAM1 3’ region was PCR amplified using the primers 5’ - ATAAggcgcgccACGAAGAAGAAACCACAAATCT - 3’ and 5’- ATAAggcgcgccAATCGGTGTATTCTTAATTAATGTCTAAAGTA - 3’ and cloned to the 3’ of the *YPET-HAM1* DNA fragment. The whole fragment was then cloned into the *pMOA34* binary vector (Barrell and Conner, 2006). *pMOA* series of binary vectors do not contain any 35S promoter elements and they have been used for generating the translational fusion fluorescence reporters (Nimchuk et al., 2011). The *pMOA34 pHAM1::YPET-HAM1* plasmid was then introduced into *ham123* triple homozygous plants through the floral dip method (Clough and Bent, 1998). To generate the *pHAM2::YPET-HAM2* in *ham123*, the *pMOA34 pHAM2::YPET-HAM2* plasmid that was described previously (Zhou et al., 2018) was introduced into *ham123* triple homozygous plants through the Agrobacterium-mediated floral dip method (Clough and Bent, 1998).

To generate the *pHAM3::YPET-HAM3* in *ham123*, a YPET-HAM3 fusion was generated using overlapping PCR with the primers 5’- TACCGAGGGTATGAATGAATTGTACAAAAAATCTAGAATGCCCTTACCCTTTGAAGAGTTTCAAGG- 3’, 5’- CCTTGAAACTCTTCAAAGGGTAAGGGCATTCTAGATTTTTTGTACAATTCATTCATACCCTCGGTA - 3’, 5’- CACCGTATTTTTACAACAATTACCAACAAC - 3’ and 5’- TCAGGAGGAGCGACATCTCCATGCT- 3’. A 3816 bp *HAM3* promoter was PCR amplified using the primers 5’- ACAAgcggccgcTTTATAAGACTTGCTATGGTCGTGAG - 3’ and 5’- ACAAgcggccgcTGCAGACGATAAAAAATAGTGTATT - 3’ (restriction enzyme sites are in lower case), and cloned to the 5’ of the *YPET-HAM3* DNA fragment. A 1755 bp *HAM3* 3’ region was PCR amplified using the primers 5’ - TACAggcgcgccTTTCCACCGGAGTTTCAATTATTAAA - 3’ and 5’- TACAggcgcgccTTAGTTGAAGGACAAATAACACCAAA - 3’ and cloned to the 3’ of the *YPET-HAM3* DNA fragment. The whole fragment was then cloned into the *pMOA34* binary vector (Barrell and Conner, 2006). The *pMOA34 pHAM3::YPET-HAM3* plasmid was then introduced into *ham123* triple homozygous plants through the floral dip method (Clough and Bent, 1998).

To generate the *pHAM2::YPET-HAM3* in *ham123*, the *HAM2* promoter was cloned to the 5’ of the *YPET-HAM3* DNA fragment and the *HAM2* 3’ region was cloned to the 3’ of the *YPET-HAM3* DNA fragment. The whole fragment was then cloned into the *pMOA34* binary vector (Barrell and Conner, 2006). The *pMOA34 pHAM2::YPET-HAM3* plasmid was introduced into *ham123* triple homozygous plants through the floral dip method (Clough and Bent, 1998).

Independent transformants were first selected based on their hygromycin resistance. They were then imaged using the laser scanning confocal microscope to determine the expression of each HAM fusion protein in the SAMs. For each construct, multiple independent transgenic lines have been identified and used in the study. Specifically, four independent transgenic lines of the *pHAM1::YPET-HAM1* in *ham123* showed comparable expression patterns of YPET-HAM1 in the SAMs and displayed comparable growth phenotypes. Three independent transgenic lines of the *pHAM2::YPET-HAM2* in *ham123* showed comparable expression patterns of YPET-HAM2 in the SAMs and displayed comparable growth phenotypes. Four independent transgenic lines of the *pHAM3::YPET-HAM3* in *ham123* showed comparable expression patterns of YPET-HAM3 in the SAMs and displayed comparable growth phenotypes. Four independent transgenic lines of the *pHAM2::YPET-HAM3* in *ham123* showed comparable expression patterns of YPET-HAM3 in the SAMs and displayed comparable growth phenotypes. The results from one representative line for each construct were presented in the Figures.

### RNA *In Situ* Hybridization Assays

All the plants for RNA *in situ* hybridization experiments were grown in short days at 22°C. Vegetative shoots were fixed with 4% paraformaldehyde overnight at 4°C. Tissues were embedded, sectioned, hybridized and washed as described previously (Krizek, 1999; Han et al., 2020b). The *CLV3* probe was described previously (Zhou et al., 2018). At least three biological replicates were performed for each genotype and showed similar results.

### Confocal Live Imaging and Quantification

The live imaging of *pHAM1::YPET-HAM1*, *pHAM2::YPET-HAM2*, *pHAM3::YPET-HAM3* or *pHAM2::YPET-HAM3* in the SAMs of *ham123* mutants was performed using the ZEISS LSM880 confocal microscope. Plants were grown in short days for three weeks and then moved to continuous light to induce flowering. The inflorescence shoot apices were imaged when plants bolted at ~2 cm in height. At least four biological replicates were imaged for each *pHAM::YPET-HAM* reporter and showed similar results. The confocal imaging was performed with the similar methods previously described (Li et al., 2013; Zhou et al., 2015; Zhou et al., 2018; Geng and Zhou, 2019). Specifically, the shoot apices were stained with PI and imaged using the W plan-Apochromat 20x/1.0 water dipping lens (Zeiss). YPET and PI were excited by a 514 nm laser line and collected from the 520-560 nm and from the 600-675 nm, respectively. In general, the signal intensities of *pHAM::YPET-HAM* translational reporters are weaker than that of the previously reported *HAM* transcriptional reporters (Zhou et al., 2015; Zhou et al., 2018). In this study, all the confocal images were taken using the sum function as the averaging method in the Zeiss ZEN software. The confocal parameters of the laser power, gain and pinhole are different when imaging different *pHAM::YPET-HAM* translational reporters. The quantification for each confocal image was performed using the Fiji software with the Fire function as previously reported (Zhou et al., 2018).

### The Modified Pseudo-Schiff Propidium Iodide (mPS-PI) Staining and Imaging

All of the plants for the mPS-PI staining (**Figure 6**) were grown in short days for 28 days at 22°C. Old leaves were dissected out from vegetative shoots, and the vegetative shoot apices were fixed and stained following the procedure previously described (Truernit et al., 2008), except that Visikol (https://visikol.com/) instead of chloral hydrate was used. The confocal images were taken using a ZEISS LSM880, and the 3D projection view for each SAM was generated and analyzed using Image J.

## Results

As previously reported, a single mutation of *HAM1*, *HAM2*, or *HAM3* does not result in any obvious defects (Engstrom et al., 2011) and the *ham123* triple loss of function mutant leads to the ectopic activation of *CLV3* in the rib meristem of SAMs (Schulze et al., 2010; Zhou et al., 2018), suggesting that *HAM1-3* likely have shared functions. To precisely define the expression pattern of each HAM protein and to evaluate the role of each HAM in shaping the *CLV3* domain, we generated HAM translational fluorescence reporters (*pHAM::YPET-HAM*) under the control of endogenous promoters and 3’ terminators, and introduced each reporter into *ham123* mutants.

**Figure 1:**
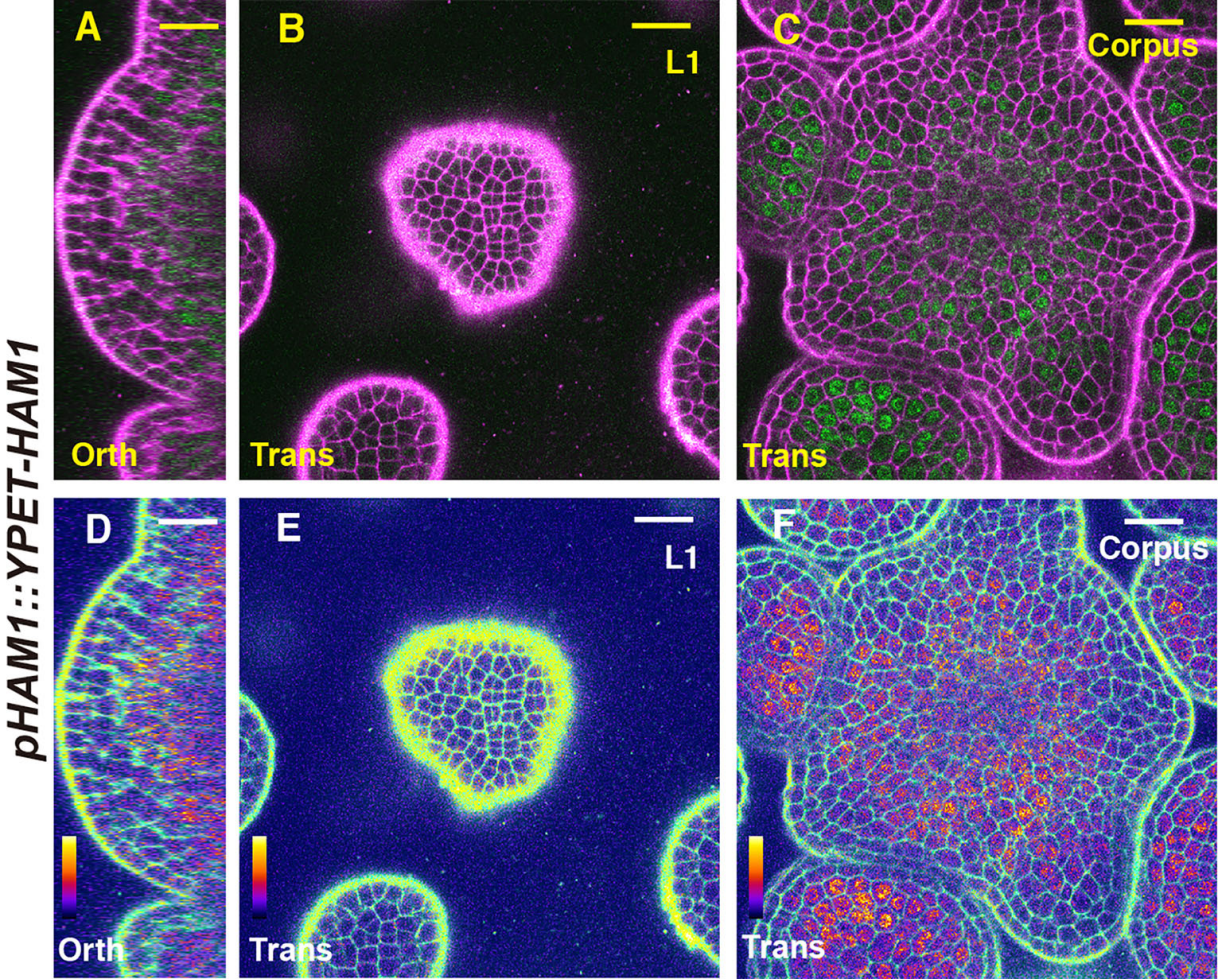
The expression of a HAM1 translational reporter in the SAM. **(A–F)** Confocal imaging of a *pHAM1::YPET-HAM1* translational reporter in a SAM of a *ham123* triple mutant, from the orthogonal view **(A, D)**, transverse optical section view in L1 **(B, E)**, and corpus **(C, F)**. **(A–C)** merged channels from YFP (green) and PI (propidium iodide, purple). **(D–F)** merged channels from the quantified YFP (quantitatively indicated by color) and PI. Scale bar: 20 µm. Color bar: Fire quantification.

**Figure 2:**
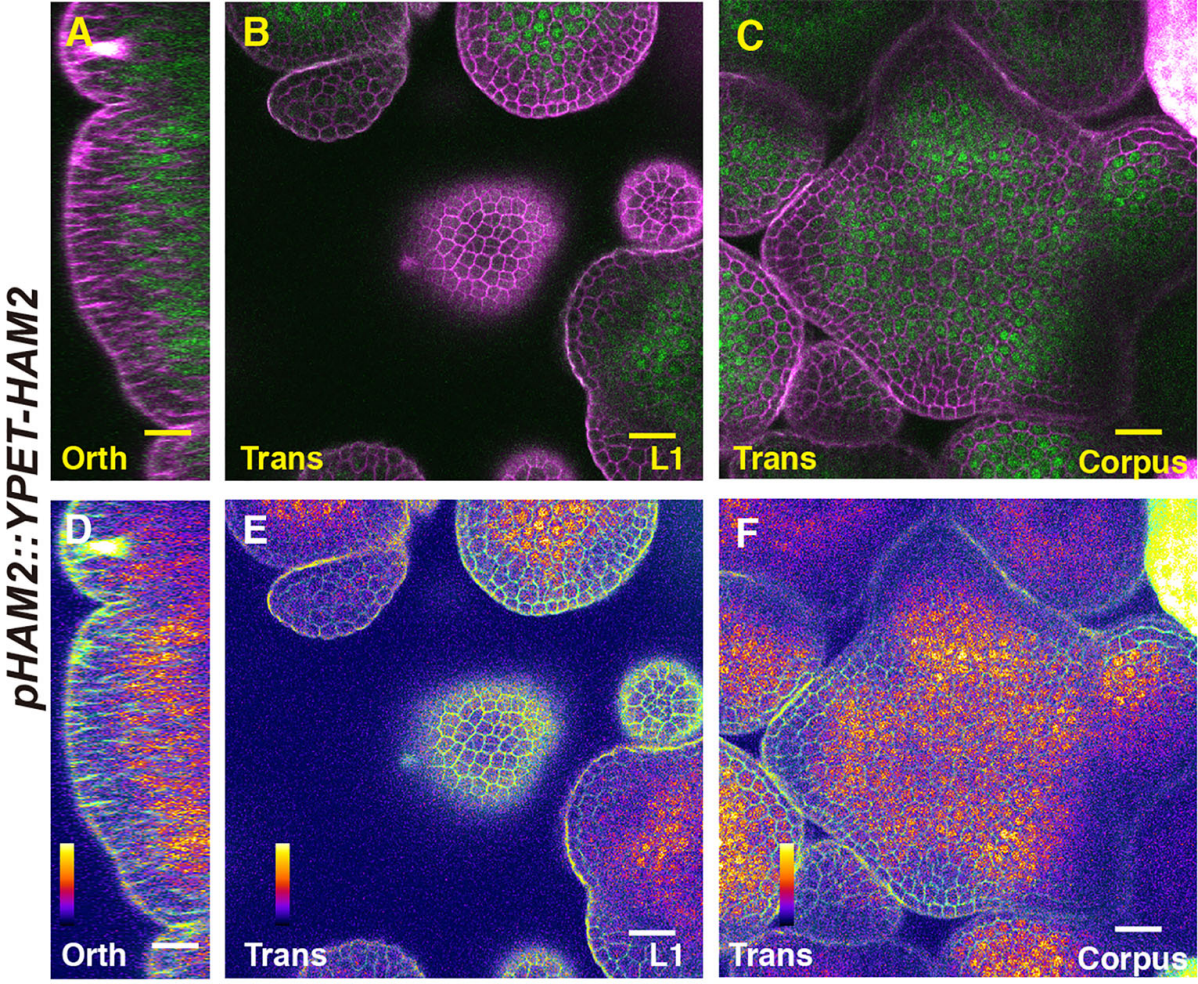
The expression of a HAM2 translational reporter in the SAM. **(A–F)** Confocal imaging of a *pHAM2::YPET-HAM2* translational reporter in a SAM of a *ham123* triple mutant, from the orthogonal view **(A, D)**, transverse optical section view in L1 **(B, E)**, and corpus **(C, F)**. **(A–C)**: merged channels from YFP (green) and PI (propidium iodide, purple). **(D–F)**: merged channels from the quantified YFP (quantitatively indicated by color) and PI. Scale bar: 20 µm. Color bar: Fire quantification.

**Figure 3:**
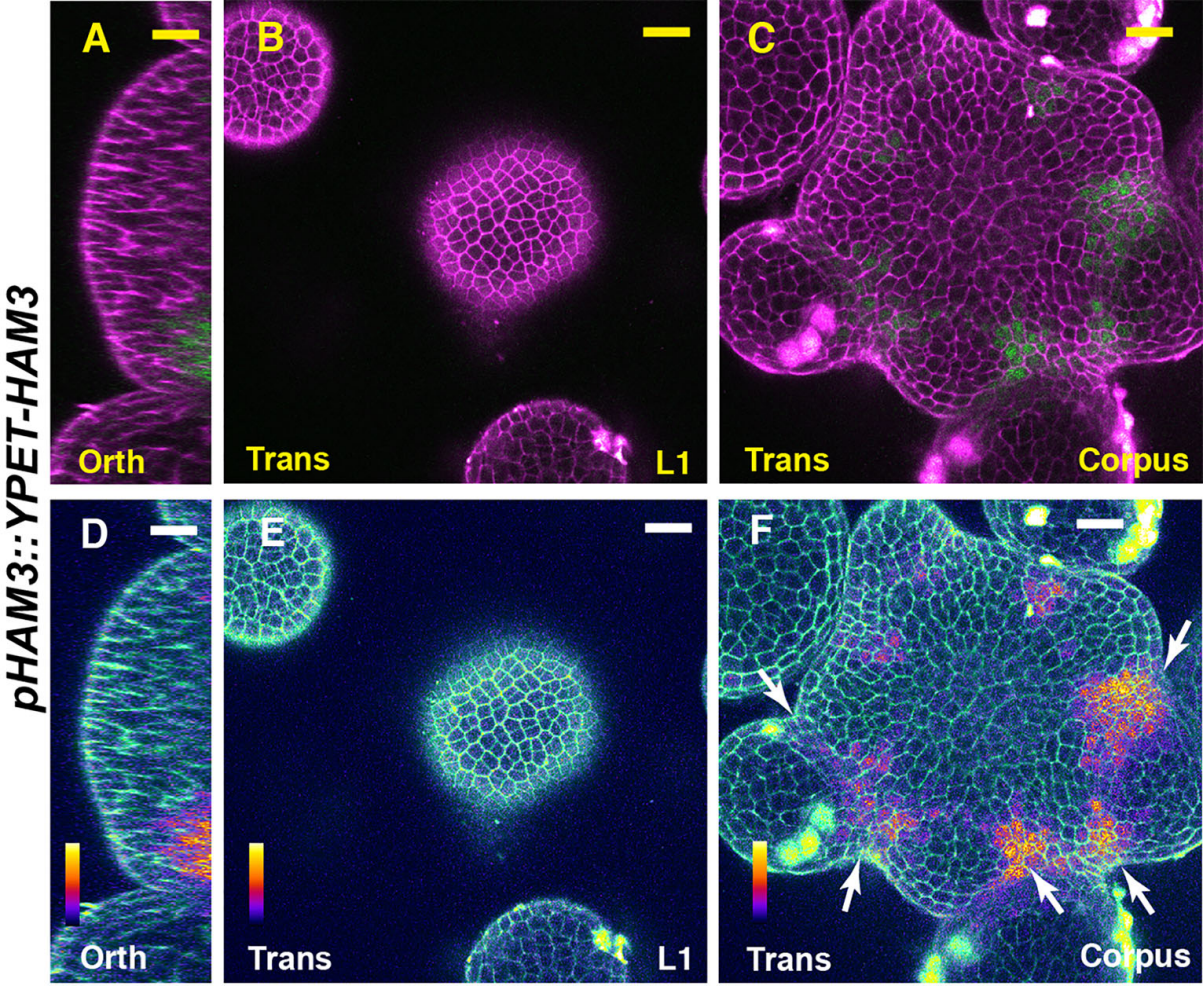
The expression of a HAM3 translational reporter in the SAM. **(A–F)** Confocal imaging of a *pHAM3::YPET-HAM3* translational reporter in a SAM of *ham123*, from orthogonal view **(A, D)**, transverse view in L1 **(B, E)**, and corpus **(C, F)**. **(A–C)**: merged channels from YFP (green) and PI (purple). **(D–F)**: merged channels from the quantified YFP (quantitatively indicated by color) and PI. Arrows indicate the boundary between shoot meristem and primordia. Scale bar: 20 µm. Color bar: Fire quantification.

We first imaged a *pHAM1::YPET-HAM1* translational reporter in the SAM from orthogonal and transverse optical section views, using confocal microscopy (**Figures 1A–C**). The signal intensity of the HAM1 translational reporter is weaker than the *HAM1* transcriptional reporter we imaged previously (Zhou et al., 2015; Zhou et al., 2018), which we found is a common difference between translational and transcriptional reporters in live imaging. The expression of *pHAM1::YPET-HAM1* shows a concentration gradient along the apical-basal axis of the SAM (**Figures 1A–F**), consistent with our previous observations on the *pHAM1::2xYPET-N7mirS* transcriptional reporter (Zhou et al., 2015; Zhou et al., 2018). Under the control of the endogenous *HAM1* promoter and 3’ terminator, the YPET-HAM1 protein is not expressed in the epidermal L1 layer, barely expressed in the L2 layer, and highly expressed in the rib meristem and peripheral zone of the corpus (**Figures 1A–F**). In parallel, we imaged the *pHAM2::YPET-HAM2* translational reporter (Zhou et al., 2015; Zhou et al., 2018; Han et al., 2020b) in the *ham123* SAM (**Figures 2A–C**). *pHAM2::YPET-HAM2* is highly expressed in the rib meristem and peripheral zone of the corpus, but it is repressed or completely off in the L1 and L2 layers (**Figures 2A–F**), a pattern comparable to that of *pHAM1::YPET-HAM1* (**Figures 1A–F**). These results suggest that HAM1 and HAM2 proteins share similar expression patterns. We also generated a translational reporter for HAM3, and imaged the *pHAM3::YPET-HAM3* in the *ham123* background (**Figures 3A–C**). In contrast to *pHAM1::YPET-HAM1* (**Figures 1A–F**) or *HAM2::YPET-HAM2* (**Figures 2A–F**), we found that the *pHAM3::YPET-HAM3* translational reporter is only expressed at the boundary between the meristem and primordia and in a few cells of the peripheral zone (PZ) of the SAM (**Figures 3A–F**). This pattern is not overlapping with the *CLV3* or the *WUS* expression domain in either wild type or in the *ham123* mutant (Schulze et al., 2010; Zhou et al., 2018). Hence, we hypothesized that in a wild type SAM, among three Type II *HAM* genes, *HAM3* would be dispensable for the regulation of *CLV3* expression due to its lack of expression in the WUS protein domain.

**Figure 4:**
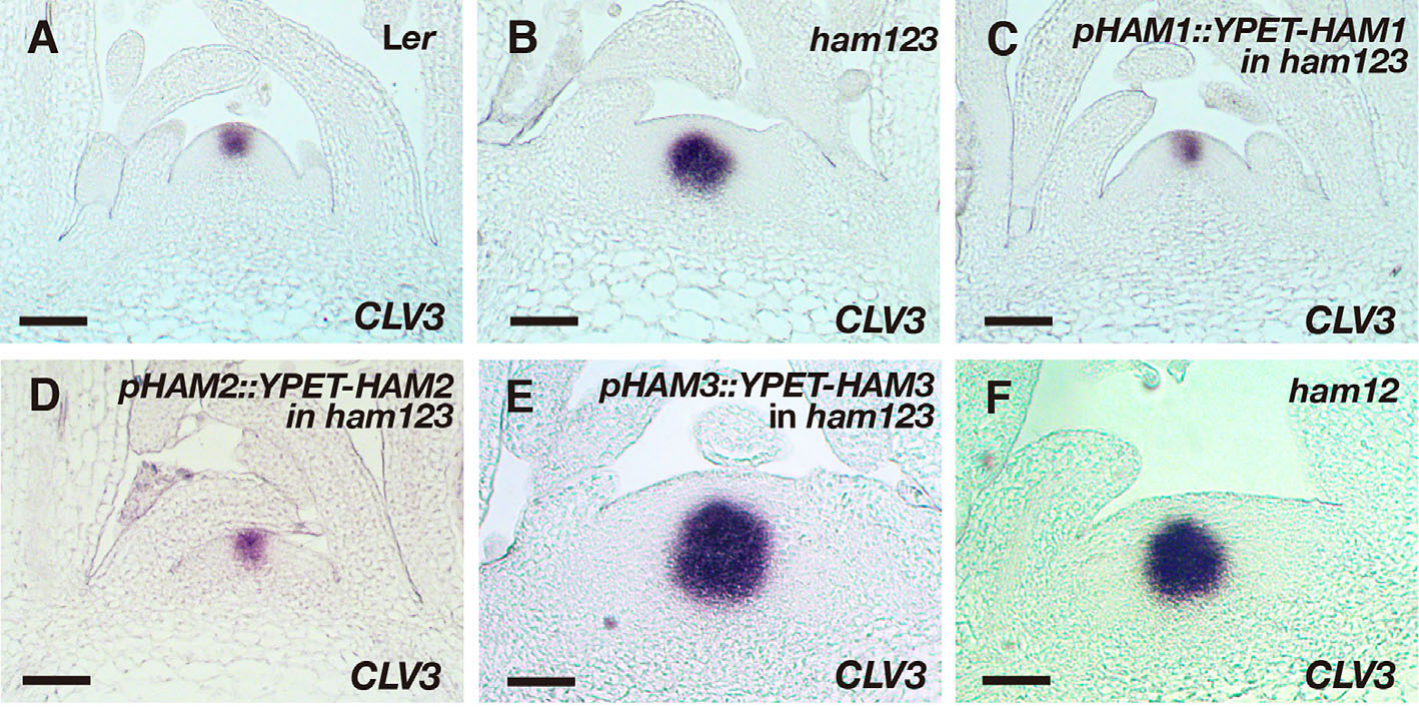
Roles of different *HAM* genes in control of *CLV3* patterning in SAMs. **(A–F)** RNA *in situ* hybridization of *CLV3* in the SAMs of wild type (L*er*) **(A)**, *ham123* **(B)**, *pHAM1::YPET-HAM1* in *ham123* **(C)**, *pHAM2::YPET-HAM2* in *ham123* **(D)**, *pHAM3::YPET-HAM3* in *ham123* **(E)**, and *ham12* **(F)** grown in short days at the same developmental stage (27 DAG). Scale bar: 50 µm. At least three biological replicates were performed for each genotype with similar results.

We then examined the expression pattern of *CLV3* using RNA *in situ* hybridization assays, in SAMs of the wild type, *ham123*, *pHAM1::YPET-HAM1 ham123*, *pHAM2::YPET-HAM2 ham123*, and *pHAM3::YPET-HAM3 ham123* plants (**Figures 4A–E**). Differently from in the wild type control, *CLV3* shows ectopic expression in the rib meristem of the *ham123* SAM (**Figures 4A, B**) (Schulze et al., 2010; Zhou et al., 2018). We found that when the SAM only expresses HAM1, in a genotype with *pHAM1::YPET-HAM1* in a *ham123* background, the *CLV3* expression domain is comparable to that in wild type (**Figure 4A**), showing a full complementation of the misregulated *CLV3* expression domain of *ham123* (**Figure 4C**). These results demonstrated that HAM1 is sufficient to keep *CLV3* expression off in the interior layers of the SAM. In the SAM of the *pHAM2::YPET-HAM2 ham123* line (**Figure 4E**), the *CLV3* expression domain was specifically confined to the central zone, comparable to a wild type (**Figure 4A**, **Figure S1**) or a *pHAM1::YPET-HAM1 ham123* plant (**Figure 4C**). The full complementation of the defective *CLV3* patterning (**Figures 4C, D****)** demonstrated that HAM2 and HAM1 share redundant function in their effect on *CLV3* expression. The RNA *in situ* hybridization results also demonstrated that the *CLV3* expression domain in *pHAM3::YPET-HAM3 ham123* plants is distinct from that in wild type and comparable to that in *ham123* (**Figure 4E**, **Figure S2**). Furthermore, we found that the expression pattern of *CLV3* in the SAMs of the *ham12* double mutants (**Figure 4F**) is largely comparable to that in the *ham123* triple mutant (**Figure 4B**, **Figures S3A–E**).

**Figure 5:**
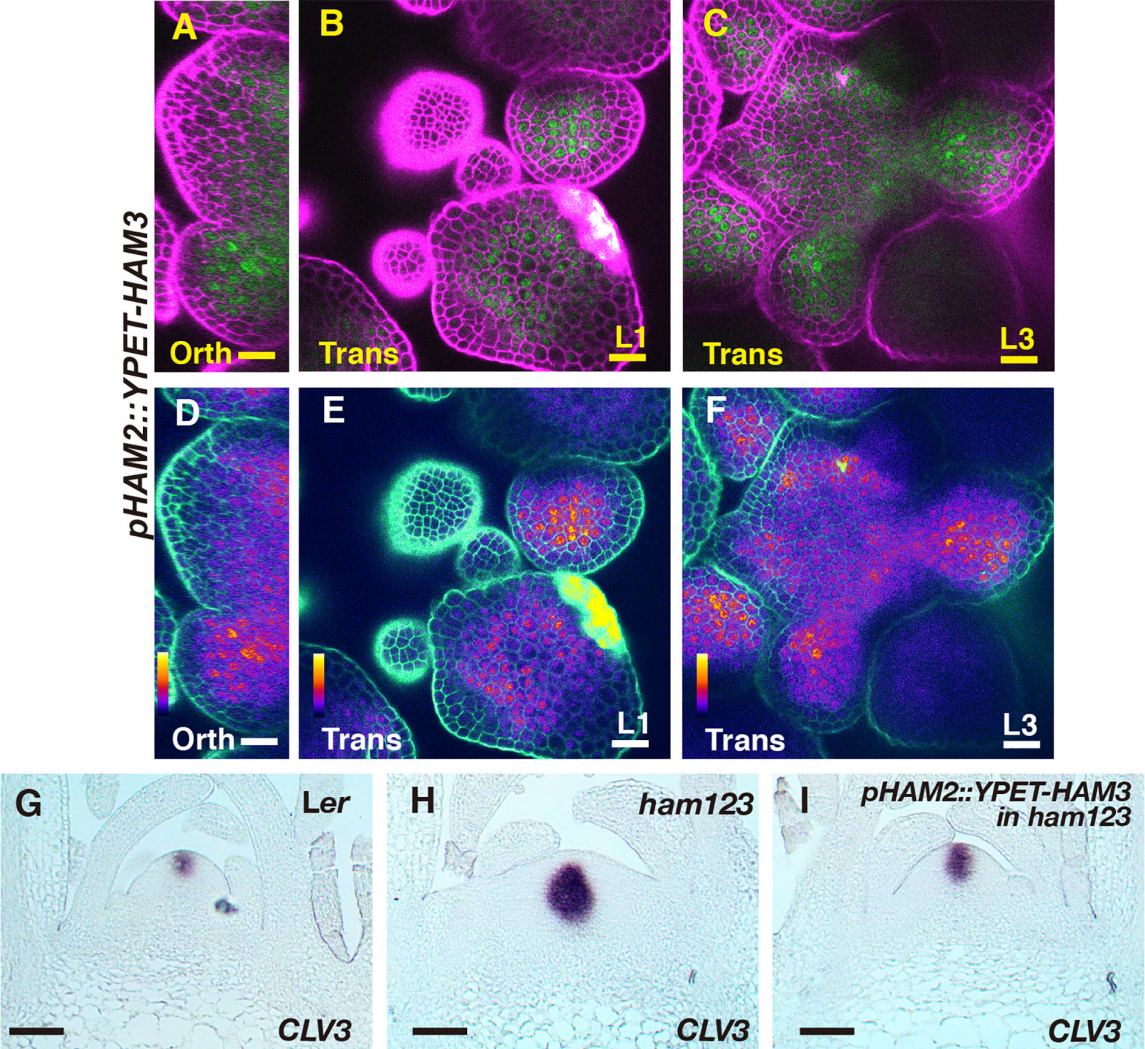
The expression and function of *pHAM2::YPET-HAM3* in the SAM. **(A–F)** Confocal imaging of a *pHAM2::YPET-HAM3* translational reporter in a SAM of *ham123* from orthogonal view **(A, D)**, transverse view in L1 **(B, E)**, and corpus **(C, F)**. **(A–C)**: merged channels from YFP (green) and PI (purple). **(D–F)**: merged channels from the quantified YFP (quantitatively indicated by color) and PI. Color bar: Fire quantification. Scale bar **(A–F)**: 20 µm. **(G–I)** RNA *in situ* hybridization of *CLV3* in the SAMs of wild type (L*er*) **(G)**, *ham123* **(H)**, and *pHAM2::YPET-HAM3* in *ham123* **(I)** at the same developmental stage (27 DAG). Scale bar **(G–I)**: 50 µm. At least three biological replicates were performed for each genotype with similar results.

The fact that *pHAM3::YPET-HAM3* does not complement the defect of *CLV3* patterning in *ham123* led us to examine whether this is because expression of the HAM3 protein is not found in the center of the rib meristem. To test this possibility, we generated a *pHAM2::YPET-HAM3* reporter in which YPET-HAM3 is under the control of the promoter and 3’ terminator of *HAM2*. We introduced this new reporter into a *ham123* triple mutant and found that the expression pattern of *pHAM2::YPET-HAM3* is largely comparable to that of *pHAM2::YPET-HAM2*, with broad expression in rib meristem and the peripheral zone in deep cell layers but reduced or absent expression in the central zone (**Figures 5A–F**). We then performed an RNA *in situ* hybridization experiment and we found that the *CLV3* expression pattern in a *pHAM2::YPET-HAM3 ham123* SAM became similar to that in the wild type plant (**Figures 5G–I**). This result suggests that the HAM3 protein maintains conserved function with HAM1/2 in regulating the *CLV3* pattern, but the endogenous *HAM3* gene alone cannot maintain correct *CLV3* patterning due to its expression domain.

**Figure 6:**
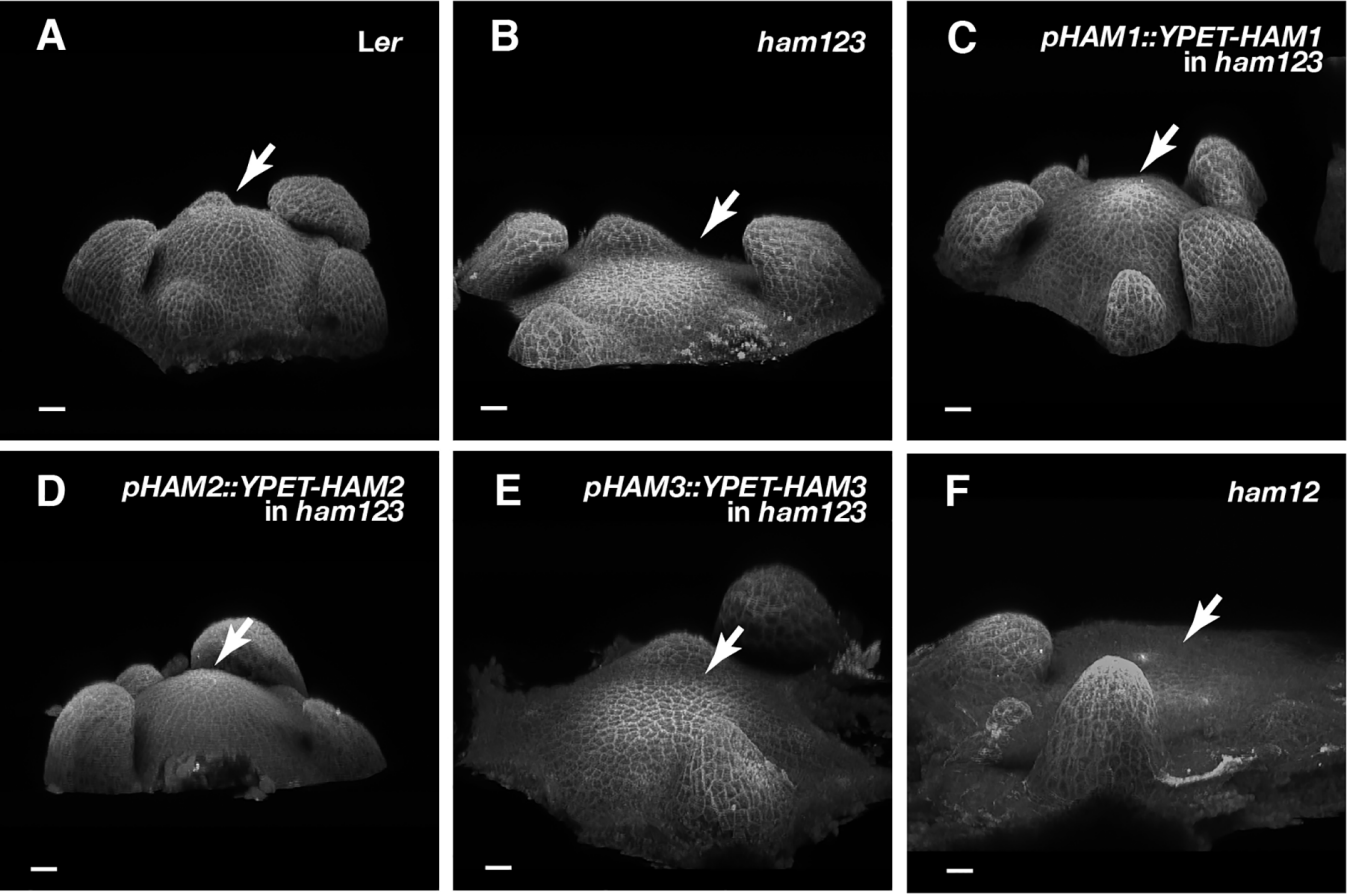
Roles of different *HAM* genes in control of vegetative SAM morphology. **(A–E)** 3D projection views of the vegetative SAMs of indicated genotypes are shown. L*er* wild type **(A)**, *ham123* **(B)**, *pHAM1::YPET-HAM1* in *ham123* **(C)**, *pHAM2::YPET-HAM2* in *ham123* **(D)**, *pHAM3::YPET-HAM3* in *ham123* **(E)**, and *ham12* **(F)** were grown in the short days and imaged at the same age (28 DAG). Four biological replicates were performed for each genotype with similar results. Arrows indicate center of the SAMs. Scale bar: 20 µm.

To further define the role of each Type II HAM member in controlling the organization of SAMs, in addition to effect on *CLV3* patterning, we imaged the SAMs of Landsberg *erecta* (L*er*) wild type, *ham123*, *pHAM1::YPET-HAM1* in a *ham123* background, *pHAM2::YPET-HAM2* in a *ham123* background, *pHAM3::YPET-HAM3* in a *ham123* background, and *ham12*, all grown in short days (**Figures 6A–F**). Similarly to previous observations (Schulze et al., 2010; Zhou et al., 2018), the SAM of a *ham123* triple mutant was flattened and broader than the dome-shaped wild type SAM (**Figures 6A, B**). Furthermore, consistent with the results from orthogonal sections in the RNA *in situ* hybridization experiments (**Figures 4C–F**), the 3D projection view of the confocal images showed that SAMs of the *pHAM1::YPET-HAM1 ham123* strain and the *pHAM2::YPET-HAM2 ham123* strain are comparable to wild type, whereas the SAMs of the *pHAM3::YPET-HAM3 ham123* strain and of *ham12* are very similar to that of *ham123* (**Figures 6C–F**).

**Figure 7:**
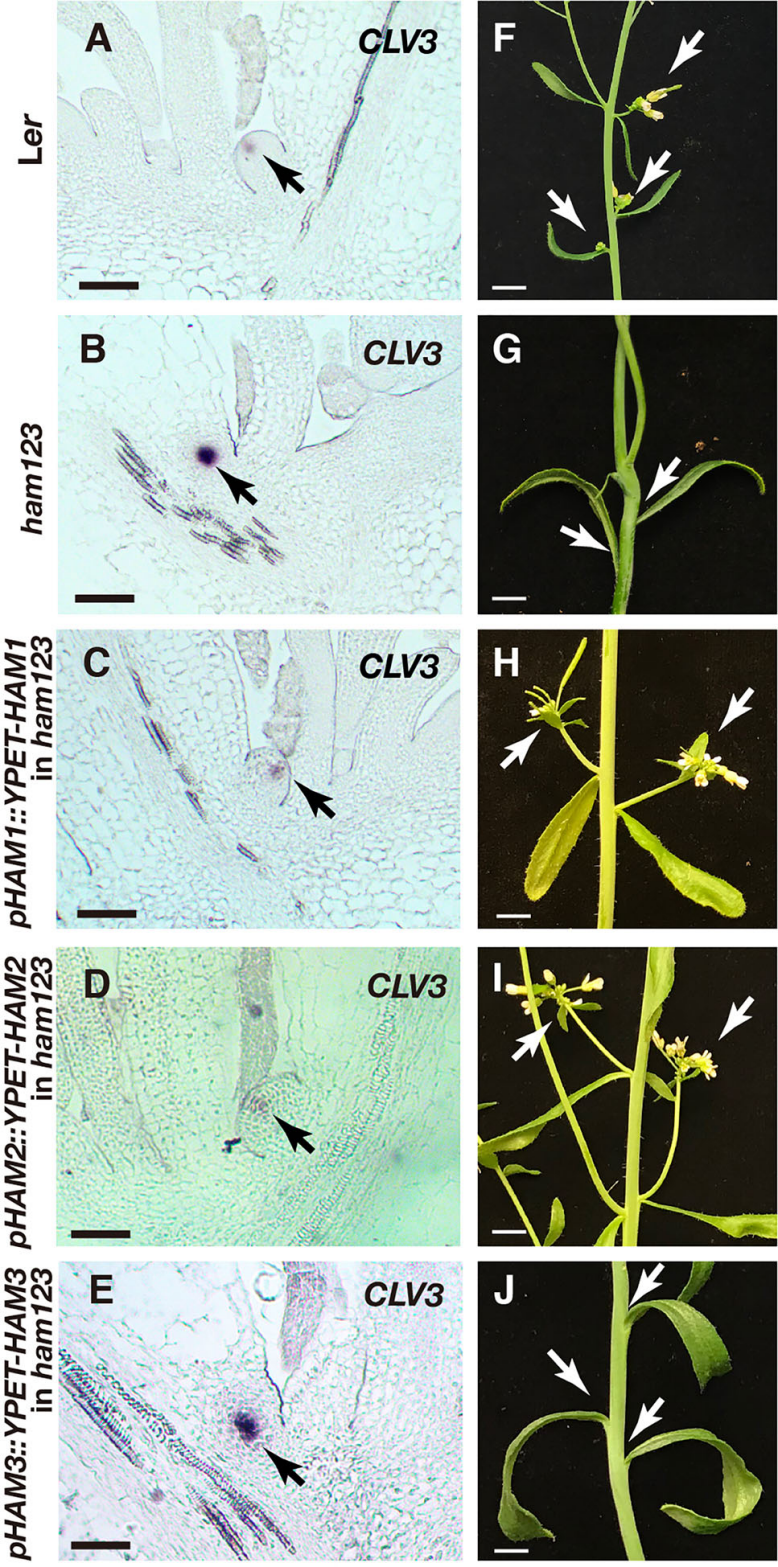
Roles of different *HAM* genes in control of *CLV3* patterning in *de novo* stem cell niches and in branch development. **(A–E)** RNA *in situ* hybridization of *CLV3* in the initiating stem cell niches of L*er* wild type **(A)**, *ham123* **(B)**, *pHAM1::YPET-HAM1* in *ham123* **(C)**, *pHAM2::YPET-HAM2* in *ham123* **(D)**, and *pHAM3::YPET-HAM3* in *ham123* **(E)**. Scale bar: 50 µm. Arrows indicate the *CLV3* expressing cells. **(F–J)** Images of branches initiated from base of the cauline leaves in different genotypes, which are grown in the same condition at the same age. Arrows indicate the branches initiated normally from the base of the cauline leaves of L*er* wild type **(F)**, *pHAM1::YPET-HAM1* in *ham123* **(H)** and *pHAM2::YPET-HAM2* in *ham123* **(I)**, and they indicate the absence of branches initiated from the base of the cauline leaves in *ham123* **(G)** and *pHAM3::YPET-HAM3* in *ham123* **(J)**. Scale bar: 0.5cm. At least three biological replicates were performed for each genotype with similar results.

In addition to established SAMs, we also examined the roles of different *HAM* genes in controlling the initiating stem cell niches in leaf axils. We previously found that *CLV3* expression is restricted to the basal part of initiating meristems in the *ham123* complete loss of function mutant (Zhou et al., 2018). Our new results show that the partial loss of function of the Type II HAM genes in a *MIR171* overexpression transgenic line is sufficient to disturb axillary meristem (AM) formation and *de novo* patterning of the *CLV3* domain (**Figures S4A–D**), and the expression of *CLV3* is also restricted to deep cell layers of the developing stem cell niches in the *MIR171 OE* line (**Figure S4D**). To further dissect the role of each *HAM* gene in controlling the initiation of stem cell niches, we examined *CLV3* expression in the initiating axillary meristems from different genotypes (**Figures 7A–E**). We found that when only HAM1 (a *pHAM1::YPET-HAM1* in *ham123* background) or HAM2 (a *pHAM2::YPET-HAM2* in *ham123* background) is present, the *CLV3* gene is expressed at the apical part of the initiating meristem (**Figures 7C, D**). In contrast, and similar to the phenotype of the *ham123* triple mutant (**Figure 7B**), the *pHAM3::YPET-HAM3 ham123* plant (**Figure 6E**) has a *CLV3* expression domain confined to deeper layers. Furthermore, the projection of the new axillary meristem from the leaf does not occur—that is, the formation of the meristem is not completed. In addition to the axillary meristems, we characterized branches initiated from the axils of cauline leaves in each genotype (**Figures 7F–J**). We found that branches can normally initiate from the cauline leaves of the wild type, the *pHAM1::YPET-HAM1 ham123* plant and the *pHAM2::YPET-HAM2 ham123* plant (**Figures 7F, H, I**). However, branches were missing from the axils of the cauline leaves in *ham123* or the *pHAM3::YPET-HAM3 ham123* plant (**Figures 7G, J**). These results in *de novo* formed meristems (**Figure 7**) together with the characterization of the primary SAM (**Figure 4**) demonstrate that HAM1 and HAM2, a pair of closely related proteins (Engstrom et al., 2011), play major roles in *de novo* patterning of *CLV3* expression in developing axillary meristems and in maintaining the *CLV3* expression domain in established SAMs.

Although the defects of meristem initiation and organization of the *CLV3* patterning in the *ham123* mutant cannot be rescued by the *pHAM3::YPET-HAM3* translational reporter at all (**Figure 4E**, **Figure 6E**, **Figures 7E, J**), the defects of retarded leaf growth in *ham123* can be largely complemented by *pHAM3::YPET-HAM3* (**Figures S5A–E**). These results suggest that HAM1/2 are necessary and sufficient for determining the *CLV3* patterns in the meristems, whereas HAM3 appears to share redundant function with HAM1/2 in control of aspects of leaf development.

## Discussion

In the previous study, we proposed and provided evidence for a model that the apical-basal extent of the *CLV3* expression domain is determined by both WUS and HAM (Zhou et al., 2018). One of the key themes in this model is that the more basally localized HAM proteins are responsible for preventing WUS induction of *CLV3* expression (Zhou et al., 2018). Here we found overlapping and distinct roles of HAM family members in control of *CLV3* patterning, closely related to their protein expression domains in the deeper cell layers of SAMs. This work supports the previously proposed WUS-HAM-CLV3 regulatory circuit (Zhou et al., 2015; Zhou et al., 2018) and further defines the cell layer-specific roles of HAM, both in established and in initiating meristems.

It has been reported that the expression patterns of the epidermis (L1)-specific marker, *ATML1* and the rib meristem (L3)-specific marker *ATHB23* (HOMEOBOX PROTEIN23) remain unaltered in a *ham123* triple mutant, comparable to that in wild type (Schulze et al., 2010). These results suggest that the ectopic expression of *CLV3* in the L3 in a *ham123* mutant is not due to a respecification of clonally distinct cell layers (from L1 to corpus/L3) in the SAMs. In line with these findings, we showed here that HAM1 and HAM2 proteins have the overlapping expression patterns in corpus/L3 (**Figures 1** and **2**) and they both are responsible for *CLV3* repression (**Figure 4**). In contrast, the endogenous HAM3 protein is dispensable for *CLV3* patterning (**Figure 4**), although it plays a role in other aspects of HAM family-mediated developmental processes (**Figure S5**). Furthermore, when the HAM3 protein is expressed in a broader region comprising L3 and RM by use of a *HAM2* promoter, it complements to a large extent the defective *CLV3* expression pattern in a *ham123* mutant (**Figure 5**). These results suggest that the specific protein expression domain is crucial for the function of HAM family members. The confocal imaging of all the *pHAM::YPET-HAM* translational reporters was performed using inflorescence SAMs. In the future, it will be important to quantitatively determine the expression patterns of these translational reporters in vegetative SAMs and in the developing AMs, to get a more comprehensive view of HAM protein localization and function during meristem development.

Differently from *WUS* and *CLV3* that are specifically expressed in a few cells, HAM1 and HAM2 are expressed in a much broader domain of the SAM (**Figures 1** and **2**) (Zhou et al., 2015), suggesting the possibility HAM1/2 have additional roles that are independent of CLV3 and/or WUS, such as regulating *STM* (Schulze et al., 2010). Future experiments will determine whether HAM1/2 can regulate the apical-basal polarity of the expression of genes other than *CLV3* and whether HAM1/2 also shape shoot architecture through CLV3-independent pathways.

## Data Availability Statement

All datasets generated for this study are included in the article/**Supplementary Material**.

## Author Contributions

HH, XL, AY, and YZ conceived the research direction. HH, YG, XL, and YZ performed the experiments. LG contributed research reagents. AY and EM discussed and commented on the experimental results. HH, XL, and YZ wrote the manuscript. AY and EM revised the manuscript. All authors contributed to the article and approved the submitted version.

## Funding

This work is supported by Purdue University start-up and funds from Purdue Center for Plant Biology to YZ. The work in EM group was funded by the Howard Hughes Medical Institute.

## Conflict of Interest

The authors declare that the research was conducted in the absence of any commercial or financial relationships that could be construed as a potential conflict of interest.

## Supplementary Material

Figure S1 Control for the RNA *in situ* experiment shown in **Figure 4D**. **(A–B)** RNA *in situ* hybridization of *CLV3* in the SAMs of L*er* wild type **(A)** and *ham123* **(B)** at the same developmental stage (27 DAG), which were grown in the identical conditions and analyzed with identical procedures together with the SAM of *pHAM2::YPET-HAM2* in *ham123* (shown in **Figure 4D**). Scale bar **(A–B)**: 50 µm.

Figure S2 Control for the RNA *in situ* experiment shown in **Figure 4E**. **(A–B)** RNA *in situ* hybridization of *CLV3* in the SAMs of L*er* wild type **(A)** and *ham123* **(B)** at the same developmental stage (27 DAG). These samples were grown in the identical conditions and analyzed with identical procedures together with the SAM of *pHAM3::YPET-HAM3* in *ham123* (shown in **Figure 4E**). Scale bar ****(A–B)****: 50 µm.

Figure S3 Roles of *HAM1* and *HAM2* in control of *CLV3* patterning in SAMs. **(A–E)** RNA *in situ* hybridization of *CLV3* in the SAMs of *ham123* **(A)** and four additional *ham12* plants (samples 2-5, **B–E)** at the same developmental stage (27 DAG). These samples were grown in the identical conditions and analyzed with identical procedures together with the SAM of *ham12* (sample 1, shown in **Figure 4F**). Scale bar **(A–E)**: 50 µm.

Figure S4 Partial loss-of function of *HAM* genes leads to the misregulation of *CLV3* in the developing AMs. **(A–D)** RNA *in situ* hybridization of *CLV3* in the AMs from wild type (L*er*) **(A–B)** and *MIR171OE* **(C–D)** at both early and late stages. Arrows indicate *CLV3* expressing cells. Scale bar: 100 µm.

Figure S5 Roles of different *HAM* genes in control of vegetative growth and leaf development. Plants with indicated genotypes, L*er* wild type **(A)**, *ham123* **(B)**, *pHAM1::YPET-HAM1* in *ham123* **(C)**, *pHAM2::YPET-HAM2* in *ham123* **(D)**, and *pHAM3::YPET-HAM3* in *ham123* were grown in the same conditions (short days) and imaged at the same age (23 DAG). Scale bar: 0.5 cm.
