# Supplementary figures and images for: The Overlapping and Distinct Roles of HAM Family Genes in Arabidopsis Shoot Meristems
Source: Front Plant Sci. 2020 Sep 4;11:541968. doi: 10.3389/fpls.2020.541968 (PMC7498855; doi:10.3389/fpls.2020.541968)

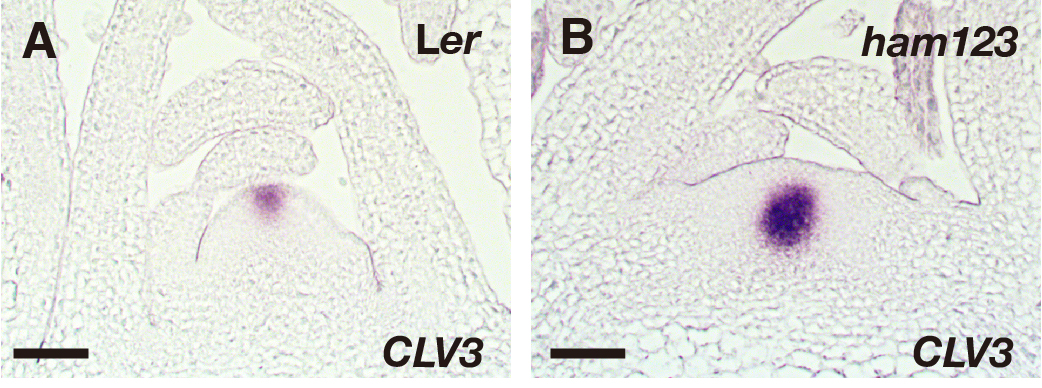

Supplement: Figure S1 — Control for the RNA in situ experiment shown in Figure 4D. (A–B) RNA in situ hybridization of CLV3 in the SAMs of Ler wild type (A) and ham123 (B) at the same developmental stage (27 DAG), which were grown in the identical conditions and analyzed with identical procedures together with the SAM of pHAM2::YPET-HAM2 in ham123 (shown in Figure 4D). Scale bar (A–B): 50 µm. [file Image_1.jpeg]

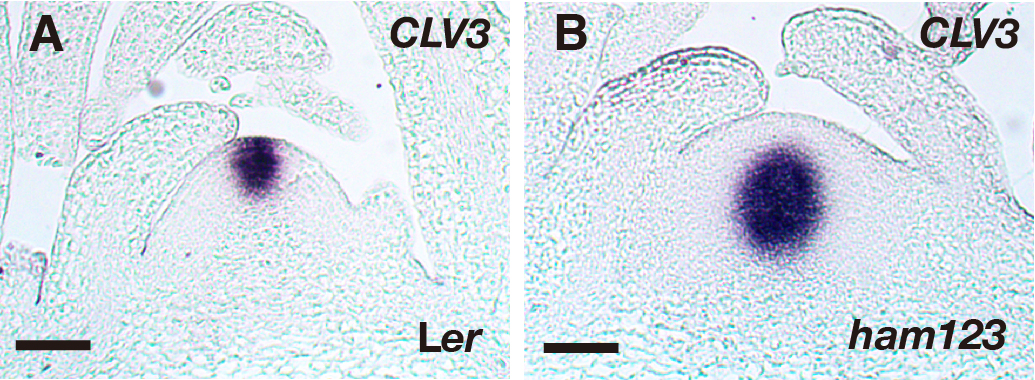

Supplement: Figure S2 — Control for the RNA in situ experiment shown in Figure 4E. (A–B) RNA in situ hybridization of CLV3 in the SAMs of Ler wild type (A) and ham123 (B) at the same developmental stage (27 DAG). These samples were grown in the identical conditions and analyzed with identical procedures together with the SAM of pHAM3::YPET-HAM3 in ham123 (shown in Figure 4E). Scale bar (A–B): 50 µm. [file Image_2.jpeg]

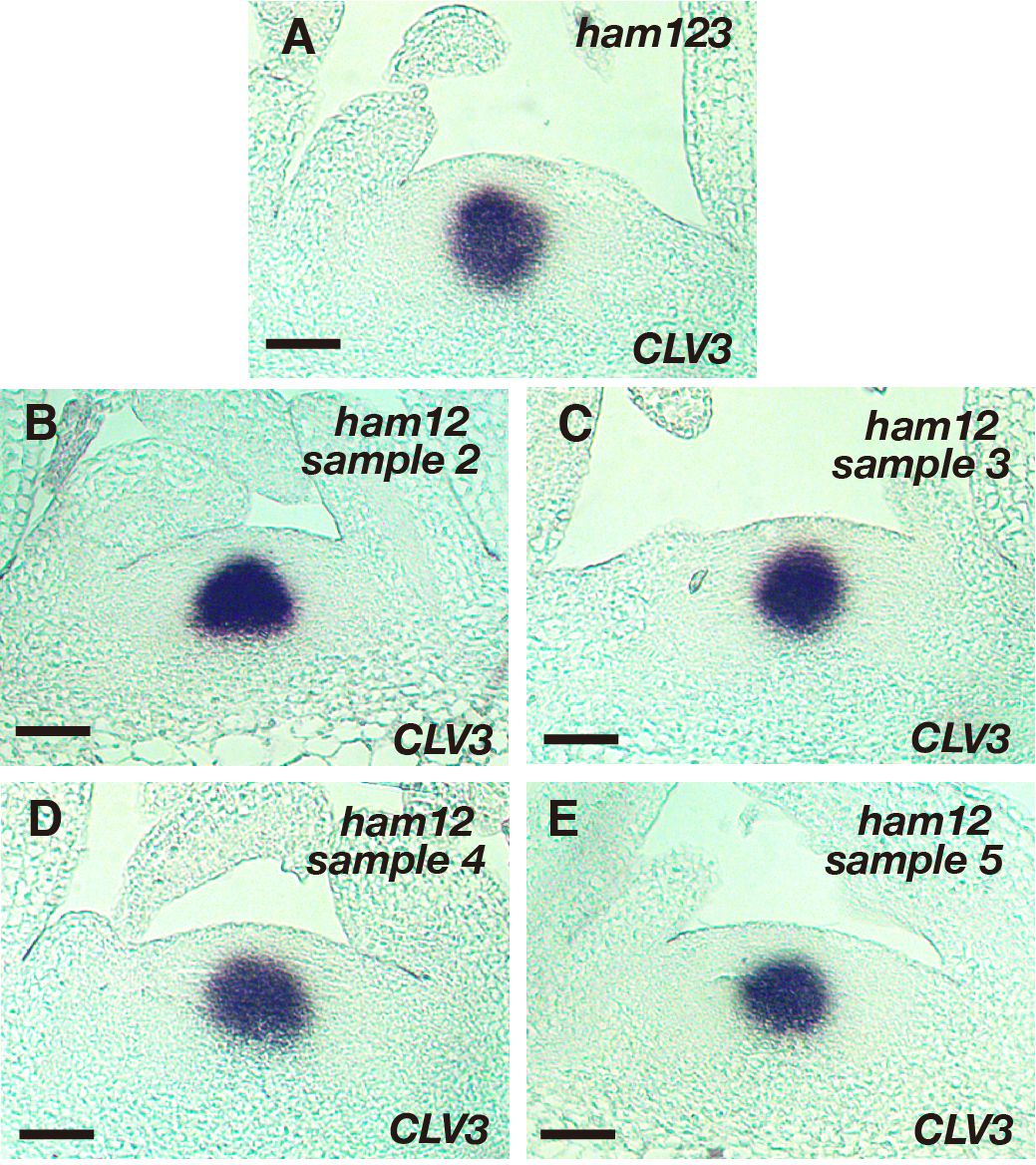

Supplement: Figure S3 — Roles of HAM1 and HAM2 in control of CLV3 patterning in SAMs. (A–E) RNA in situ hybridization of CLV3 in the SAMs of ham123 (A) and four additional ham12 plants (samples 2-5, B–E) at the same developmental stage (27 DAG). These samples were grown in the identical conditions and analyzed with identical procedures together with the SAM of ham12 (sample 1, shown in Figure 4F). Scale bar (A–E): 50 µm. [file Image_3.jpeg]

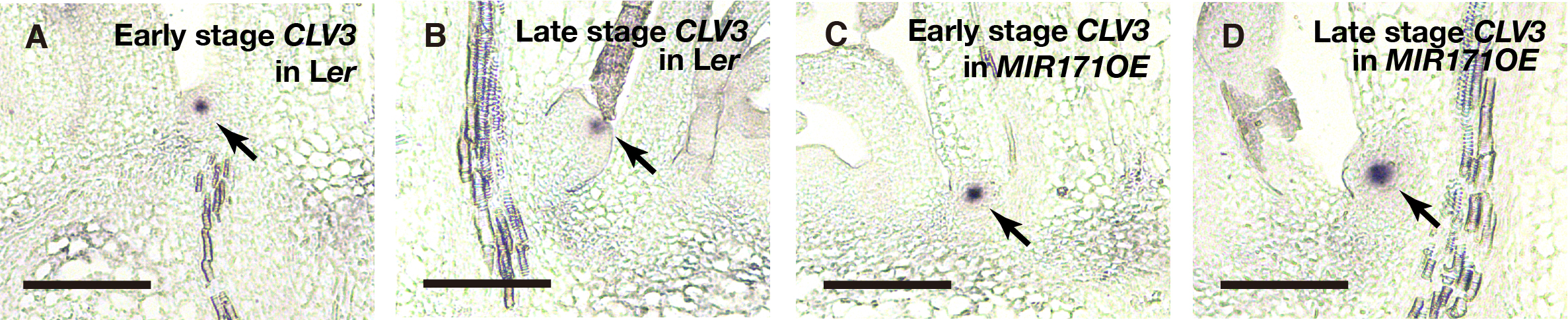

Supplement: Figure S4 — Partial loss-of function of HAM genes leads to the misregulation of CLV3 in the developing AMs. (A–D) RNA in situ hybridization of CLV3 in the AMs from wild type (Ler) (A–B) and MIR171OE (C–D) at both early and late stages. Arrows indicate CLV3 expressing cells. Scale bar: 100 µm. [file Image_4.jpeg]

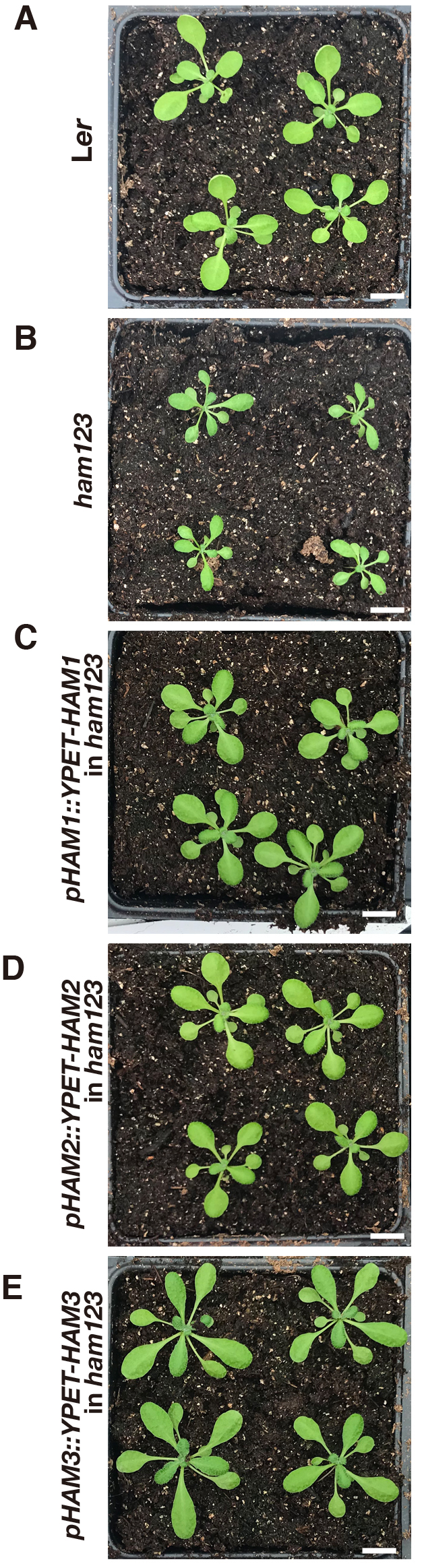

Supplement: Figure S5 — Roles of different HAM genes in control of vegetative growth and leaf development. Plants with indicated genotypes, Ler wild type (A), ham123 (B), pHAM1::YPET-HAM1 in ham123 (C), pHAM2::YPET-HAM2 in ham123 (D), and pHAM3::YPET-HAM3 in ham123 were grown in the same conditions (short days) and imaged at the same age (23 DAG). Scale bar: 0.5 cm. [file Image_5.jpeg]
